# Lactic acid bacteria as a cell factory for riboflavin production

**DOI:** 10.1111/1751-7915.12335

**Published:** 2015-12-21

**Authors:** Kiran Thakur, Sudhir Kumar Tomar, Sachinandan De

**Affiliations:** ^1^ Division of Dairy MicrobiologyNational Dairy Research InstituteKarnalHaryanaIndia; ^2^ Animal Biotechnology CentreNational Dairy Research InstituteKarnalHaryanaIndia

## Abstract

Consumers are increasingly becoming aware of their health and nutritional requirements, and in this context, vitamins produced *in situ* by microbes may suit their needs and expectations. B groups vitamins are essential components of cellular metabolism and among them riboflavin is one of the vital vitamins required by bacteria, plants, animals and humans. Here, we focus on the importance of microbial production of riboflavin over chemical synthesis. In addition, genetic abilities for riboflavin biosynthesis by lactic acid bacteria are discussed. Genetically modified strains by employing genetic engineering and chemical analogues have been developed to enhance riboflavin production. The present review attempts to collect the currently available information on riboflavin production by microbes in general, while placing greater emphasis on food grade lactic acid bacteria and human gut commensals. For designing riboflavin‐enriched functional foods, proper selection and exploitation of riboflavin‐producing lactic acid bacteria is essential. Moreover, eliminating the *in situ* vitamin fortification step will decrease the cost of food production.

## Introduction

Riboflavin is present in many foods such as green vegetables, dairy products, eggs and meat. The recommended daily intake for riboflavin is 1.3 mg day^−1^ for men and 1.1 mg day^−1^ for women (Food and Nutrition Board, 1998). In Western countries, mostly milk and dairy products contribute to the daily intake of riboflavin besides yeast, cereals, meats, fatty fish and green leafy vegetables (Cooperman and Lopez, 1991; Powers *et al*., 1993). Grain products contain only low amounts of riboflavin because of loss of this vitamin during processing of the grains. Nevertheless, fortification practices make certain breads and cereals very good sources of riboflavin (Hill and Nalubola, 2002; Powers, 2003). In defiance of the presence of most of the vitamins in a variety of foods, human riboflavin deficiency persists in both developing and industrialized countries (O'Brien *et al*., 2001; Blanck *et al*., 2002) because of insufficient food intake and unbalanced diet (LeBlanc *et al*., 2011). In developing nations, its deficiency prevails in populations whose diet lacks dairy products and meat (Combs, 1992; Rohner *et al*., 2007). A high prevalence of poor riboflavin status has been observed among adolescent girls in the United Kingdom and among the Irish population (O'Brien *et al*., 2001; Powers, 2003). Riboflavin deficiency is associated with impaired vision, reduced growth rate, increased levels of homocysteine with consequent cardiac risk (Moat *et al*., 2003), pre‐eclampsia (Wacker *et al*., 2000), oxidative stress (Ashoori and Saedisomeolia, 2014) and anaemia (Shi *et al*., 2014). Riboflavin deficiency can lead to liver and skin damage, and changes in cerebral glucose metabolism (LeBlanc *et al*., 2011) with symptoms like hyperaemia, sore throat, oedema of oral and mucous membranes, cheilosis and glossitis (Wilson, 1983).

Riboflavin has been traditionally synthesized for food and feed fortification by chemicals means, but past decade has witnessed emerging information about commercial completive microbial processes for its production (Stahmann *et al*., 2000). Riboflavin is synthesized by many bacteria and its biosynthesis pathway has been studied in both Gram‐positive and Gram‐negative bacteria, but it has been extensively studied only in two organisms namely in *Bacillus subtilis* (Perkins and Pero, 2002) and *Escherichia coli* (Bacher *et al*., 1996). Currently, three microorganisms are exploited for riboflavin production: *Ashbya gossypii*, *Candida famata* and *B. subtilis* (Perkins *et al*., 1999; Stahmann *et al*., 2000; Schallmey *et al*., 2004). In recent years, the use of lactic acid bacteria (LAB) was proposed for vitamin synthesis. These microorganisms are able to synthesize B‐group vitamins particularly riboflavin to obtain fermented bio‐enriched food (Capozzi *et al*., 2011; Laino *et al*., 2012; Vaesken *et al*., 2012). The use of LAB is a common practice in the dairy industry, and the addition of the riboflavin‐producing strain into fermented products such as fermented milks, yoghurt, and cheeses increases riboflavin concentrations, which is feasible and economically viable (LeBlanc *et al*., 2005a). The obvious practical advantages of vitamin‐producing LAB that fortification happens *in situ*. The *in situ* fortification advantage of LAB makes them a good choice for bio prospecting bacteria which can act as vitamin supplier to human hosts (Burgess *et al*., 2009). The adaptability of LAB to fermentation processes, their biosynthetic capability and metabolic versatility are the key features that make them ideal candidates for *in situ* production of riboflavin in food (Arena *et al*., 2014b). Gut commensals are able to synthesize vitamin K as well as most of the water‐soluble B‐vitamins, such as biotin, folates, nicotinic acid, panthotenic acid, pyridoxine, riboflavin and thiamine (Hill, 1997). In this review, focus is placed on the LAB and their genetic ability to biosynthesize riboflavin.

## Microbes taking place of chemical factories for riboflavin production

**Figure 1:**
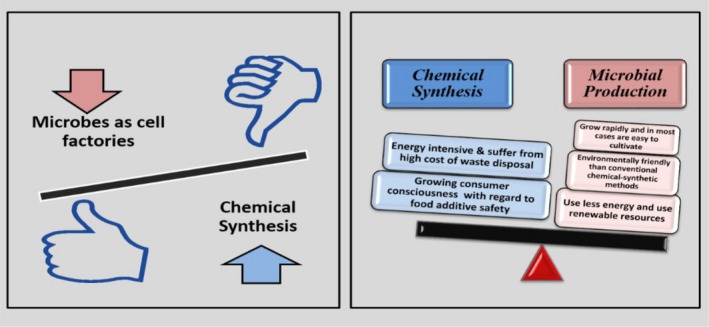
Advantages of microbes as cell factories for vitamin synthesis.

Although humans and animals lack the ability to synthesize most of the vitamins, bacteria have inherent potential to produce those metabolites (LeBlanc *et al*., 2011). With modern lifestyle, consumers are becoming more health conscious and discerned in their food choices (Burgess *et al*., 2004). In such a situation, riboflavin‐supplying LAB offer a clear advantage over chemical synthesis by increasing the nutritional value of food (LeBlanc *et al*., 2012). Chemical synthesis of a vitamin is being replaced by fermentation processes because of economic and environmental considerations of the latter. Besides the economic advantages, additional benefits of the microbial synthesis include the use of renewable sources, environmental‐friendly approach and superior quality of the final product (Fig. 1) (Van Loon *et al*., 1996).

## Importance of riboflavin to humans and bacteria

Each B‐group vitamin acts in synergy to maintain the body's homeostasis by playing major roles in metabolic processes (LeBlanc *et al*., 2011). One of such essential vitamins, i.e. riboflavin, is an obligatory component of cellular metabolism and is responsible for normal development, growth, reproduction, lactation, physical performance of well‐being. Metabolically, riboflavin is the precursor of flavin mononucleotide (FMN) and flavin adenine dinucleotide (FAD), both of which act as electron carriers in oxidation‐reduction reactions (Fischer and Bacher, 2005). They help in the metabolism of carbohydrates, amino acids, energy production and also activate folate and pyridoxine to their respective coenzyme forms (Food and Nutrition Board, 1998; Massey, 2000), which constitute the basis for its clinical applications. Until now, riboflavin has received relatively little attention, but interest is increasing with its recognition as an essential component of cellular biochemistry (Thakur and Tomar, 2015). One study has suggested that dietary vitamin intake leads to a relatively low risk of vitamin deficiency in all age and sex groups (Mensink *et al*., 2013). Riboflavin is being used for headache (Schetzek *et al*., 2013) and migraine management (Sherwood *et al*., 2014). According to Foley and colleagues (2014) riboflavin supplementation can combat the progression of neurodegenerative conditions. Another study by Shi and colleagues (2014) showed that inadequate riboflavin intake was associated with an increased risk of persistent anaemia. Riboflavin can act as a protectant from oxidative injury independently by the conversion of its reduced form to oxidized form, or as a component of glutathione redox cycle (Ashoori and Saedisomeolia, 2014). According to Hassan and colleagues (2013) riboflavin acts as an efficient adjuvant, which is confirmed in many cancer cell lines and animal‐based studies, and it is promising under photodynamic therapy (Hassan *et al*., 2013. Recently, riboflavin has been shown to improve the efficiency of conventional therapies in different diseases such as *Staphylococcus aureus* infection and cisplatin‐induced intestinal epithelial cell apoptosis (Bodiga *et al*., 2012; Mal *et al*., 2013).

Although requirement for riboflavin may be rare among bacteria, it is known to be an essential growth factor for *Enterococcus faecalis*, *Streptococcus pyogenes*, *Listeria monocytogenes* and some lactobacilli (Koser, 1968). The biosynthetic deficiency correlates with the absence of riboflavin biosynthetic genes in the genomes of these organisms (Vitreschak *et al*., 2002). The sensitive growth response of *Lactobacillus casei* to riboflavin was used to develop one of the first microbiological assays for a vitamin (Snell and Strong, 1939) and it is based on the presence of an efficient transport system that allows the uptake of exogenous riboflavin. Riboflavin uptake inversely correlates with the riboflavin concentration present during cell growth and increases in riboflavin‐requiring mutants (Coquard *et al*., 1997).

## Regulation of riboflavin biosynthesis in bacteria

**Figure 2:**
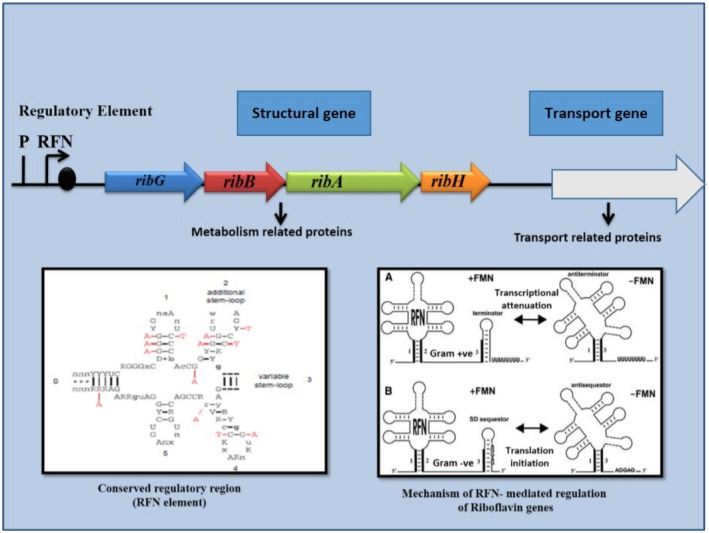
Regulation of riboflavin biosynthesis genes in Gram‐positive and Gram‐negative bacteria.

**Figure 3:**
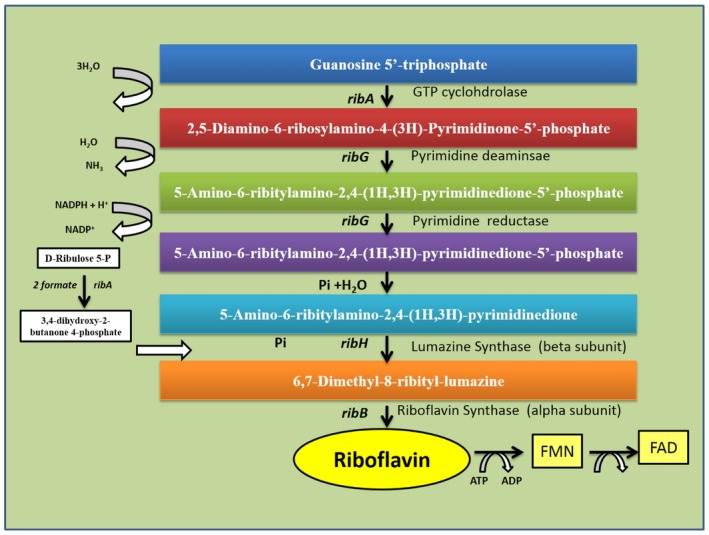
Riboflavin biosynthesis pathway in bacteria.

The riboflavin biosynthesis in bacteria was analysed using comparative analysis of genes, operons and regulatory elements (Vitreschak *et al*., 2002). A model for regulation of riboflavin biosynthesis is based on the formation of alternative RNA structure involving the RFN element (a mononucleotide riboswitch is highly conserved RNA element that is found frequently in the 5′ untranslated region of prokaryotic mRNA that encodes for FMN biosynthesis and transport proteins) (Fig. 2) (Gelfand *et al*., 1999; Vitreschak *et al*., 2002). The RFN element can be found on the chromosome of many, but not all, bacterial species (Gelfand *et al*., 1999; Vitreschak *et al*., 2002; Wels *et al*., 2006). In Gram‐positive bacteria, riboflavin metabolism and transport genes are regulated at transcription attenuation, whereas in Gram‐negative bacteria riboflavin biosynthesis genes are regulated on level of translation initiation (Fig. 2) (Vitreschak *et al*., 2002). The enzymatic activities required to catalyse the biosynthesis of riboflavin from guanosine triphosphate (GTP) and ribulose‐5‐phosphate are encoded by four genes (*ribG, ribB, ribA* and *ribH*) as shown in (Fig. 3) (Perkins *et al*., 1999). According to these authors, these genes are located in an operon, the gene order of which differs from the order of enzymatic reactions. In GTP *cyclohydrolase II* activity, which catalyses the first step in riboflavin biosynthesis, GTP is encoded by the third gene in the operon, *ribA*. The *RibA* gene also contains a second enzymatic function that synthesizes a four‐carbon unit from ribulose‐5‐phosphate (Richter *et al*., 1992; 1993) that is utilized in a later step (*lumazine synthase*). The second and third enzymatic steps (deamination of the pyrimidine ring of structure and the subsequent reduction of the ribosyl side‐chain) are controlled by another bi‐functional enzyme encoded by the first gene of the operon *ribG* (Richter *et al*., 1997). The penultimate step in riboflavin biosynthesis, is catalysed by *lumazine synthase*, the product of the last *rib* gene, *ribH* (Perkins *et al*., 1999). Riboflavin synthase, which controls the last step of the pathway, is encoded by the second gene of the operon, *ribE* (Perkins *et al*., 1999). Transcription of the four riboflavin genes is primarily controlled by the *ribP1* promoter and regulatory region located at the 5′ end of the operon (Perkins *et al*., 1999). In addition, the last two *rib* genes in the operon, *ribA* and *ribH*, are also transcribed from a second promoter (*ribP*2) and regulatory region RFN (Perkins *et al*., 1999).

## Genetic ability of riboflavin production in LAB

According to Capozzi and colleagues (2012), the genetic information for riboflavin biosynthesis in LAB is species specific and/or strain specific. It is clear from the previous reports of comparative genome analysis that the ability to synthesize riboflavin is shared by several of the sequenced members of LAB although an interrupted or partially present *rib* operon is sometimes observed in certain strains (Table 1). According to bioinformatics analysis by Burgess and colleagues (2004), when the first gene (*ribG)* is absent from the genome, it is more likely that the riboflavin operon will be incomplete. The sequenced genome of *Lactobacillus plantarum* strain WCFS1 contains an incomplete *rib* operon, which is devoid of the entire *ribG* and part of the *ribB* genes (Kleerebezem *et al*., 2003). As expected, this strain is unable to grow in the absence of riboflavin (Burgess *et al*., 2006).

**Table 1:** Presence/absence of riboflavin biosynthesis genes among different LAB strains (adapted from Capozzi *et al*., 2012 and Valle *et al*., 2014)

Organism	*rib*G	*rib*B	*rib*A	*rib*H
*Lactococcus lactis* subsp. *cremoris* SK11	+	+	+	+
*Lactococcus lactis* subsp. *cremoris* NZ9000	+	+	+	+
*Lactococcus lactis* subsp. *lactis* KF147	+	+	+	+
*Lactococcus lactis* subsp. *cremoris* A76	+	+	+	+
*Lactococcus lactis* subsp. *cremoris* MG1363	+	+	+	+
*Lactococcus lactis* subsp. *lactis* CV56	+	+	+	+
*Lactobacillus brevis* ATCC367	+	+	+	+
*Lactobacillus plantarum* WCFSI	−	−	+	+
*Lactobacillus plantarum* subsp. *plantarum* ST‐III	+	+	+	+
*Lactobacillus plantarum* JDM1	+	+	+	+
*Lactobacillus plantarum* CRL725	+	+	+	+
*Lactobacillus gasseri* ATCC33323	−	−	−	+
*Lactobacillus casei* ATCC334	−	−	−	−
*Lactobacillus bulgaricus* ATCC BAA365	−	−	−	−
*Lactobacillus delbrueckii* subsp. *bulgaricus* ND02	+	+	+	+
*Lactobacillus delbrueckii* subsp*. bulgaricus* 2038	+	+	+	+
*Lactobacillus delbrueckii* subsp*. bulgaricus* ATCC 11842	−	−	−	−
*Lactobacillus acidophilus* NCFM	−	−	−	−
*Lactobacillus acidophilus* 30SC 8293	−	−	−	+
*Leuconostoc mesenteroides* subsp. *mesenteroides* J18	+	+	+	+
*Leuconostoc mesenteroides* subsp. *mesenteroides* ATCC 8293	+	+	+	+
*Lactobacillus amylovorus* GRL1118	−	−	−	+
*Lactobacillus amylovorus* GRL 1112	−	−	−	+
*Lactobacillus buchneri* NRRL B‐30929	−	−	−	−
*Lactobacillus crispatus* ST1	+	+	+	+
*Lactobacillus fermentum IFO 3956*	+	+	+	+
*Lactobacillus fermentum MTCC8711*	+	+	+	+
*Lactobacillus helveticus DPC 4571*	−	−	−	−
*Lactobacillus helveticus* H10	−	−	−	−
*Lactobacillus johnsonii* NCC 533, DPC 6026, FI9785	−	−	−	−
*Lactobacillus kefiranofaciens* ZW3	−	−	−	−
*Lactobacillus reuteri* DSM 20016	+	+	+	+
*Lactobacillus reuteri* SD2112	−	−	+	−
*Lactobacillus reuteri JCM 1112*	+	+	+	+
*Lactobacillus rhamnosus* ATCC 8530, GG	−	−	−	−
*Lactobacillus salivarius* CECT 5713, UCC118	+	+	+	+

+, Presence; −, absence.

## Human gut commensals and riboflavin biosynthesis ability

According to a recent study of systematic genome assessment of B‐vitamin biosynthesis, a complete riboflavin operon is present in all *Bacteroidetes*, *Fusobacteria* and 36 genomes (92%) of *Proteobacteria* (Magnusdottir *et al*., 2015). In their study, the authors have placed *Firmicutes* as more potent producers of riboflavin compared with other examined vitamins. The *Actinobacteria* phylum contains only two genomes that are publicly available, namely, those of *Corynebacterium ammoniagenes* DSM 20306 and *Bifidobacterium longum* ATCC 15697, which have the coding capacity for riboflavin biosynthesis. Interestingly, gut commensals that produce riboflavin are detected by the innate immune system through a metabolic intermediate as riboflavin precursors found in many bacteria and yeast selectively activating mucosal‐associated invariant T cells, an abundant population of innate‐like T cells in humans (Corbett *et al*., 2014). Riboflavin biosynthesis genes seem to be partially or completely absent from the majority of currently available bifidobacterial genomes (Ventura *et al*., 2007). Recently, four bifidobacterial species are predicted to possess a complete riboflavin biosynthesis pathway (Milani *et al*., 2014), which may represent an additional mechanism for microbe–host interactions by stimulation of the host's immune system (Corbett *et al*., 2014). The possibility of co‐evolution of gut microbes in the human gut makes them suitable for *de novo* synthesis (LeBlanc *et al*., 2012; Magnusdottir *et al*., 2015). The latter study suggests that human gut bacteria actively exchange B‐vitamins among each other, which leads to the survival of organisms that do not synthesize any of these essential cofactors. However, all non‐producing organisms from the human gut contained the riboflavin transporter role, indicating their need for the riboflavin‐derived cofactors FMN and FAD. It was almost completely absent in the *Bacteroidetes*, *Fusobacteria* and *Proteobacteria*, whereas the de novo synthesis pathway was found in nearly all genomes of the three phyla (Magnusdottir *et al*., 2015). Another study supports the fact that vitamin metabolism pathways are highly represented in all enterotypes, whereas two among all the examined enterotypes are found to be rich in biosynthesis genes for biotin, riboflavin, pantothenate, ascorbate, thiamine and folate production (Arumugam *et al*., 2011).

## Riboflavin production by LAB

LAB are a group of industrially prominent microorganisms used in the food and dairy industry because of their enormous applications for the biosynthesis of a number of compounds as metabolic end‐products or secondary metabolites (LeBlanc *et al*., 2012). Many LAB and bifidobacteria produce a range of metabolites including B‐vitamins such as riboflavin and folate, low‐calorie sugars such as mannitol and sorbitol, exopolysaccharides, diacetyl and L‐alanine (Hugenholtz *et al*., 2002). They also accumulate and biotransform inorganic selenium to organic and elemental forms, which are useful for human and animal nutrition. (Pophaly *et al*., 2014; Saini *et al*., 2014). There are three reports of riboflavin‐producing lactobacilli from India (Table 2) (Jayashree *et al*., 2010; Guru and Viswanathan, 2013; Thakur and Tomar, 2015). Thakur and Tomar, (2015) have reported the riboflavin production in *Lactobacillus fermentum* KTLF1 (2.36 mg) and *L. plantarum* (and 2.13 mg l^−1^) in MRS medium (Thakur and Tomar, 2015). According to Jayashree and colleagues (2010) efficient riboflavin‐producing bacterium *L. fermentum* MTCC 8711 showed 2.29 mgl l^−1^ of riboflavin in chemically defined media after 24 h. Guru and Viswanathan (2013) have observed that *L. acidophilus* produces higher riboflavin levels compared with *Lactococcus lactis*. They have recommended whey as a better fermentation medium compared with skim milk for riboflavin production (Guru and Viswanathan, 2013). Valle and colleagues (2014) have evaluated over 179 strains of LAB to increase the riboflavin levels in soymilk. The development of novel functional foods with enhanced vitamin content has been suggested and it would contribute to an ever‐growing market for these products (Stanton *et al*., 2005). The production of fermented food products with elevated levels of B‐vitamins increases their commercial and nutritional value and eliminates the need for fortification (Burgess *et al*., 2009). Different strategies have been applied to improve microbial production of vitamins during fermentation (Sybesma *et al*., 2006). Riboflavin overproduction can be achieved either by genetic engineering (Perkins *et al*., 1991) or by exposure to purine analogues and/or the toxic riboflavin analogue roseoflavin (Table 2) (Burgess *et al*., 2004). The same authors have obtained overproduction of riboflavin up to 24 mg l^−1^ and up to around 0.9 mg l^−1^ using nisin induction and roseoflavin respectively (Burgess *et al*., 2004). Often, the increased riboflavin production phenotype is associated with mutations at the regulatory region (RFN), which increases the transcription of the riboflavin operon (Burgess *et al*., 2006). Roseoflavin‐resistant strains of *Leu. mesenteroides* overproduced up to 0.5 mg l^−1^ of riboflavin, whereas riboflavin‐overproducing *L. plantarum* and *Propionibacterium freudenreichii* were able to synthesize up to around 0.6 mg l^−1^ and 3 mg l^−1^ respectively (Burgess *et al*., 2006). The genetic engineering is an interesting way to exploit the industrially important strains that cannot produce riboflavin or in other strains that produce it at low level and are physiologically inactive (Capozzi *et al*., 2012). These two strategies for riboflavin overproduction have been successfully employed in various LAB and non‐LAB so far.

**Table 2:** Various LAB and non‐LAB screened for riboflavin production

Riboflavin production strategy	Organism	Source	References
Genetic engineering/exposure to purine/toxic riboflavin analogue	Microbes screened for enhanced riboflavin production		
	*L. lactis*	Yoghurt	LeBlanc and colleagues (2005)
	*L. lactis* subsp. *cremoris* strain NZ9000		Burgess and colleagues (2004)
	*L. fermentum* MTCC8711		Jayashree and colleagues (2011)
	*L. plantarum*		Burgess and colleagues (2006)
	*L. mesenteroids*		Burgess and colleagues (2006)
	*P. freudenreichii*		Burgess and colleagues (2006)
Exposure to toxic riboflavin analogue	*L. plantarum*, *L. mesenteroides* and *L. fermentum*	Sourdough	Russo and colleagues (2014)
	*L. plantarum* CRL725	Dairy products	Valle and colleagues (2014)
	*L. plantarum*	Durum wheat flour	Capozzi and colleagues (2011)
Natural	Microbes screened for natural riboflavin production		
	*L. acidophilus*	Curd and cheese	Guru and Viswanathan, 2013
	*Bacillus clausii*, *B. subtilis*, *B. cereus* IP 5832, *L. rhamnosus* ATCC 53103	Probiotic formulations	Salvetti *et al*., 2003
	*L. fermentum*, *L. plantarum* and *L. mucosae*	Human faeces and fermented bamboo shoots	Thakur and Tomar, 2015
Genetic engineering/exposure to purine/toxic riboflavin analogue	Commercial producers		
	*A. gossypii*		Perkins and colleagues (1999)
	*Candida famata*		Schallmey and colleagues (2004)
	*Bacillus subtilis*		Stahmann and colleagues (2000)

## Riboflavin overproduction by genetic engineering approach

*Lactococcus lactis* is a commonly used starter strain that can be converted from riboflavin consumer into riboflavin‐producing factory by overexpressing its riboflavin biosynthesis genes (LeBlanc *et al*., 2005b). These riboflavin‐producing strains were able to eliminate most physiological manifestations of ariboflavinosis such as stunted growth, elevated erythrocyte glutathione reductase activation coefficient values and hepatomegalia in a riboflavin depletion–repletion model. In another study, Burgess and colleagues (2004) carried out genetic analysis of the riboflavin biosynthetic operon in *L. lactis* subsp. *cremoris* strain NZ9000. The strain showed enhanced vitamin synthesis because of simultaneous overexpression of riboflavin biosynthetic genes (*ribG*, *ribH*, *ribB* and *ribA*) in *L. lactis* (Burgess *et al*., 2004). In one study, the inactivation of the *folE* gene, involved in the folate biosynthesis pathway, which could make more GTP available for the riboflavin biosynthesis, resulted in a 50% enhanced level of riboflavin production by *L. fermentum*, albeit with double generation time. This phenotype was stably maintained because the *folE* has been disrupted in the genome (Jayashree *et al*., 2011). While through site‐directed mutagenesis followed by metabolic engineering, Sybesma and colleagues (2004) modified two complicated biosynthetic pathways in *L. lactis* that resulted in simultaneous overproduction of both folate and riboflavin (Sybesma *et al*., 2004). Such strategies do not attempt to generate alternative production strains, but rather replacing riboflavin‐consuming strains used in traditional food fermentation processes with riboflavin‐producing counterparts, thereby increasing riboflavin bioavailability in the food product and introducing an added health benefit. Elevated levels of the vitamin, which would be produced in such foods, would not have any negative health implications as no upper limit of intake has been set for riboflavin because of the lack of evidence on adverse effect in humans (Flynn *et al*., 2003).

## Riboflavin overproduction by chemical analogues approach

The isolation of spontaneous roseoflavin‐resistant mutants is a reliable method to obtain natural riboflavin‐overproducing strains of various species commonly used in the food industry, and it is also acceptable from a consumer/regulatory point of view as it does not involve deliberate genetic engineering (Jayashree *et al*., 2011). With the increase in the availability of genome sequences it is possible not only to identify the potential mutations that cause riboflavin (over) production, but also to determine how stable such mutations are maintained (LeBlanc *et al*., 2012). The toxic analogue approach has also been successfully employed for *L. plantarum*, *Leuconostoc mesenteroides* and *P. freudenreichii* (Burgess *et al*., 2006) and a fermented dairy product made with the latter strain was shown to counteract riboflavin deficiency in an animal model (LeBlanc *et al*., 2006). The riboflavin‐overproducing *Lactobacillus* strains were selected by exposure to roseoflavin and several overproducing strains were identified and used for bread fermentation, barley and oat‐fermented products (Russo *et al*., 2012).The riboflavin‐producing LAB strains including *L. plantarum*, *L. mesenteroides* and *L. fermentum* were isolated from a traditional sourdough (Russo *et al*., 2014). Overproducing strains of *L. fermentum* and *L. plantarum* selected after exposure to roseoflavin were investigated for their probiotic attributes by using an *in vitro* model and they were able to synthesize riboflavin in co‐culture systems with Caco‐2 cells (Arena *et al*., 2014a). It was reported that β‐Glucans stimulate the growth of these strains when submitted to oro‐gastrointestinal stress with a positive impact on bacterial adhesion (Arena *et al*., 2014b). Moreover, the adhesion ability of these strains was evaluated by using gnotobiotic zebrafish larvae as *in vivo* model, reinforcing the suggestion that they could contribute to further increase the riboflavin supply in the gut environment (Russo *et al*., 2015). The *in vitro* adhesion on human epithelial cell lines (mucus‐producing HT‐29) was also studied by the use of riboflavin‐producing *L. mucosae* KTF (Thakur *et al*., 2015). Russo and colleagues (2014) have used *L. fermentum* PBCC11.5 and its parental strain to fortify bread, and they have concluded that bread produced using the co‐inoculum yeast and *L. fermentum* PBCC11.5 led to an approximately twofold increase of final riboflavin content, which opens new perspectives in the field of functional foods based on a cereal matrix (Russo *et al*., 2014). In one study by LeBlanc and colleagues (2006) the novel fermented product containing *P. freudenreichii* B2336, with increased levels of riboflavin, eliminated most physiological manifestations of ariboflavinosis using a riboflavin depletion–repletion model, whereas the product fermented with the non‐riboflavin‐producing strain did not show this beneficial effect. *Propionibacterium freudenreichii* NIZO B2336 is a spontaneous roseoflavin‐resistant mutant derived from *P. freudenreichii* B374 that produces higher levels of the riboflavin than that produced by the parental stain (LeBlanc *et al*., 2006). In another study, riboflavin‐producing LAB strains were isolated and used as a convenient biotechnological application for the preparation of fermented sourdough bread and pasta to enrich them with riboflavin (Capozzi *et al*., 2011). In this study, *L. plantarum* was selected for roseoflavin‐resistant to acquire natural riboflavin‐overproducing strains. Valle and colleagues (2014) stated that roseoflavin‐resistant strains are capable of synthesizing riboflavin in soymilk and have led to an interesting and economically feasible biotechnology strategy that could easily be adapted to develop novel vitamin bio‐enriched functional foods with enhanced consumer appeal. All these reports (Table 3) of enhanced riboflavin production in various dairy and cereal‐based products pave the way for analysing the effect of similar riboflavin‐overproducing LAB in human trials (LeBlanc *et al*., 2005).

**Table 3:** *In vivo* manifestations of riboflavin‐enriched fermented products and riboflavin‐overproducing lactobacilli

Product	Organism used	*In vivo* effects	Reference
Fermented milk	*P. freudenreichii* B2336	Eliminated most physiological manifestation of ariboflavinosis	LeBlanc and colleagues (2006)
Fermented milk	*L. lactis* NZ9000	Reversing ariboflavinosis in a riboflavin‐deficiency rat model	LeBlanc and colleagues (2005)
–	*L. lactis* CB010	Elimination of stunted growth, increased EGRAC values and hepatomaglia in animal model riboflavin depletion–repletion rats	–
Soya milk	*L. plantarum* CRL 725	–	Valle and colleagues (2014)
Yoghurt	*P. freudenreichii* B2336	–	Burgess and colleagues (2004)
Pasta and bread	*L. plantarum*	–	Capozzi and colleagues (2011)

EGRAC, erythrocyte glutathione reductase activity coefficient.

## Probiotics and B‐vitamin biosynthesis

Besides traditional applications of LAB, some of the members have been reported to elicit probiotic features (Russo *et al*., 2015). Food‐related LAB as well as human gut commensals such as bifidobacteria make a certain site in dairy and food industry by imparting various health benefits to human host and carries enzymes to *de novo* synthesize and supply vitamins (LeBlanc *et al*., 2012). Two sources of riboflavin are available to humans: a dietary source and riboflavin‐producing microflora of the large intestine (Wrong *et al*., 1981; Hill, 1997). Vitamins produced by microbes get adsorbed in the colon in contrast to dietary vitamins, which are adsorbed in the proximal tract of the small intestine (Ichihashi *et al*., 1992; Said and Mohammed, 2006). The site of uptake increases the bioavailability of vitamins synthesised by microbes to human host. Moreover riboflavin‐producing gut commensals may overactivate the innate immune system (Corbett *et al*., 2014), which also presents the limitations of *in situ* riboflavin production by gut commensals. Commercialized probiotic bacteria have been included as active ingredients in products such as yoghurt, cheese, ice cream, chocolates pharmaceutical tablets, infant formulas and dietary supplements (Tamime *et al*., 2005). Fermented foods using LAB are advantageous because they have the potential beneficial effects of probiotic properties coupled with enhanced content of vitamins (Jayashree *et al*., 2011). Intestinal microbiota has also been shown to produce short chain fatty acids, conjugated linoleic acid, essential amino acids, group B‐vitamins and vitamin K, contributing to the well‐being of a host (Marques *et al*., 2010). According to Magnusdottir and colleagues (2015) gut microbiota is an important source of B‐vitamins, which lead to changes in the gut microbiota composition and ultimately affecting our dietary B‐vitamin requirements. Salvetti and colleagues (2003) have reported eight probiotic strains from five different probiotic formulations containing *Bacillus clausii*, *B. subtilis*, *Bacillus cereus* IP 5832, *Lactobacillus rhamnosus* ATCC 53103, which were able to produce riboflavin (Salvetti *et al*., 2003). Guru and Viswanathan (2013) have reported riboflavin‐producing probiotic *L. acidophilus* obtained from curd and cheese samples.

## Concluding comments

The economic and environmental considerations have led the fermentation‐based method as a model of the environmentally friendly white biotechnology with regard to traditional chemical synthesis of riboflavin (Shi *et al*., 2009). Bacteria producing even small amounts of riboflavin will be a better choice to be used as a starter for the formation of fermented products rather than traditional starters, which consume riboflavin. So far, information available on whole genomes of various microbes has made it clear that riboflavin‐producing ability is recognized to be strain or subspecies specific. Thus, it can be an attractive approach to bioprospect prolific riboflavin‐producing strains from their diversified natural niche and further enhance their ability to produce this essential vitamin by microbiological and biotechnological interventions. The enzymes required for riboflavin biosynthesis may be completely or partially absent in various available genomes of microbes; nevertheless, the behaviour of multiple coexisting microbial species suggests the possibility of *de novo* synthesis of riboflavin. LAB also known as power house of dairy industry and imparting health benefits as probiotics are endowed with the ability to synthesize essential biomolecules in particular riboflavin. The carefully selected riboflavin‐producing strains holding probiotic attributes could open the way to be potential candidates for *in situ* production of riboflavin once these strains get colonized to host intestine (Arena *et al*., 2014a, 2014b). Thus, considering the extensive application of LAB in the food, pharmaceutical and medicine industry, coupled with consumer demand for healthier foods, the use of these food grade microorganisms as riboflavin factories will be of great advantage in the near future.

## Conflict of interest

None declared.
